# The role of walnut bZIP genes in explant browning

**DOI:** 10.1186/s12864-023-09492-1

**Published:** 2023-07-05

**Authors:** Hui Wang, Jiali Peng, Yaoling Li, Lishan Xu, Wenqiang Dai, Shugang Zhao

**Affiliations:** 1 College of Life Sciences, Hebei Agricultural University, Baoding, 071001 China; 2 College of Horticulture, Hebei Agricultural University, Baoding, 071001 China

**Keywords:** Walnut, bZIP, Explant, Browning

## Abstract

**Background:**

Basic leucine zipper (bZIP) proteins are important transcription factors in plants. To study the role of bZIP transcription factors in walnut explant browning, this study used bioinformatics software to analyze walnut bZIP gene family members, along with their transcript levels in different walnut tissues, to evaluate the transcriptional expression of this gene family during the primary culture of walnut explants and to reveal the mechanism of action of walnut *bZIP* genes in walnut explant browning.

**Results:**

The results identified 65 *JrbZIP* genes in the walnut genome, which were divided into 8 subfamilies and distributed on 16 chromosomes. The results of transcriptome data analysis showed that there were significant differences in the expression of four genes, namely, *JrbZIP55, JrbZIP70, JrbZIP72,* and *JrbZIP88*, under both vermiculite and agar culture conditions. There were multiple hormone (salicylic acid, abscisic acid, auxin, and gibberellin) signaling and regulatory elements that are responsive to stress (low temperature, stress, and defense) located in the promoter regions of *JrbZIP55, JrbZIP70, JrbZIP72,* and *JrbZIP88*. The walnut JrbZIP55 protein and *Arabidopsis* bZIP42 protein are highly homologous, and the proteins interacting with *Arabidopsis* bZIP42 include the AT2G19940 oxidoreductases, which act on aldehyde or oxygen-containing donors.

**Conclusion:**

It is speculated that JrbZIP55 may participate in the regulation of browning in walnut explants.

**Supplementary Information:**

The online version contains supplementary material available at 10.1186/s12864-023-09492-1.

## Background

*bZIP* (basic leucine zipper) genes encode one of the largest and most highly conserved families of transcription factors and repressor proteins in eukaryotes [1]. *bZIP* proteins in plants generally contain 60 to 80 amino acid residues and include 2 structural units: a highly conserved DNA binding region and a variable leucine zipper dimer region [2]. DNA structural regions typically contain 16 amino acid residues with a highly conserved N-X7-R/K motif. The leucine zipper is typically characterized by 1 leucine per 7 amino acids as well as hydrophobic amino acids at the 3rd and 4th positions [3]. To date, *bZIP* genes have been identified in the genomes of various plants, including *Arabidopsis* [4], maize [5], tomato [6], rice [2], apple [7], kiwi [8], date palm [9], tobacco [10] and wheat [11], and it has been shown that this family of genes is involved in plant biotic and abiotic stress responses [6, 12], seed germination [13], anthocyanin accumulation [14], lignin synthesis [15] and the regulation of growth and developmental processes [4] and that *bZIP* transcription factors participate in plant responses to ABA, light and developmental signals by regulating functional gene expression [16, 17]. The main function of *bZIP* transcription factors is to regulate the intensity of target gene expression in response to exogenous hormones and environmental stresses [16]. Under stressful environmental conditions, *bZIP* transcription factors can regulate the expression of downstream target genes by binding to the promoter regions of ABA-inducible genes, and ABA signaling plays a crucial role in plant responses to abiotic stress, such as defense against pathogens or the control of spatiotemporally specific expression of target genes [18].

Browning, also known as the browning reaction, is a phenomenon that occurs in all life stages of a plant and results in the creation of a brown polymer through a series of reactions [19]. Plant tissues produce large amounts of unstable phenolics that are secreted after damage, and the formation of brown quinones from these phenolics is then catalyzed by a variety of oxidative enzymes. This enzymatic browning is the main cause of explant browning [20]. Browning can lead to the death of explants in tissue culture, affect the experimental process, reduce the efficiency of plant genetic transformation, and reduce the quality of fruits by browning during postharvest transport [21]. Therefore, the study of the effects of plant browning inhibition is particularly important. Some studies have found that overexpression of *StSPI128* significantly alleviated potato browning and increased phenolic content [22]. Browning inhibition in mushrooms can be achieved by using nano-PM to inhibit TYR activity, which in turn delays the conversion of GHB to GDHB and tyrosine to DOPA, ultimately reducing melanogenesis [23], and the addition of ascorbic acid to DKW medium significantly reduced the browning of the medium and the explants [19]. Aloe vera gel can be used as an edible coating (dry environment) and as a gel solution (aqueous environment: a new method) to prevent browning during the refrigeration of fresh Persian walnut kernels [24]. However, the regulatory mechanism of plant tissue browning needs to be further investigated.

*Juglans regia* L., also known as English walnut, is a deciduous tree of the genus Walnut in the family Walnutaceae. It is native to southeastern Europe, western Asia and southwestern China [25]. China has a long history of walnut cultivation and ranks first in the world in terms of the cultivated area and production of walnut [25]; however, because walnuts are rich in polyphenols, their explants are highly susceptible to browning, and the difficulty of seedling rooting has become a limiting factor in walnut factory nurseries [26]. In recent years, with the surge in demand for woody food and oil plants, the demand for walnut seedlings in domestic and international markets has also expanded each year [27]. Therefore, overcoming explant browning and speeding up the process of factory seeding are important issues in the walnut industry. Higher compatible solute levels in the leaves of in vitro plants do not contribute to water conservation during ex vitro acclimation, and low foliar ion levels in in vitro plants can occur due to the low transpiration rate of plants as a result of in vitro production [28]. Walnut tissue is rich in phenolic substances [29], causing it to brown particularly easily after injury. It is also susceptible to browning during tissue culture. Thus, we used vermiculite as the primary medium, which effectively reduced the browning rate of explants, mainly because of the air permeability of vermiculite, which can mitigate damage to the explants, resulting in a high survival rate [30]. Studies have shown that *bZIP* genes in tomato are involved in defense responses during tissue damage [6], but whether *bZIP* genes are also involved in regulatory processes during the culture of walnut explants is unclear. Therefore, this study was conducted to mine and identify the bZIP family members of the walnut genome and analyze the changes in the expression of *bZIP* genes during explant culture. The aim was to clarify the regulatory role of the *bZIP* gene family in tissue damage and to lay the foundation for resolving the mechanism of browning in walnut explants.

## Results

### Identification and basic information of bZIP gene family members of walnut

Hidden Markov models were constructed using *Arabidopsis* bZIP family protein sequence information, *bZIP* genes in the walnut genome were searched using the Hmmer tool, and 91 walnut *bZIP* family genes were screened and tentatively named *JrbZIP1-JrbZIP91*. Using the Pfam database and the results of prediction analysis with online SMART software, after the removal of redundancy [31], 65 walnut *bZIP* transcription factor family gene members were finally identified [32]. The physical and chemical properties of the walnut bZIP family members were analyzed (Table 1), and the results showed that the theoretical isoelectric point (PI) of *JrbZIP* ranged from 4.85 (*JrbZIP72*) to 10.04 (*JrbZIP59*), gene length ranged from 453 bp (*JrbZIP51*) to 23704 bp (*JrbZIP58*), CDS length ranged from 399 bp (*JrbZIP49*) to 2352 bp (*JrbZIP70*), relative molecular masses ranged from 15.08 kD (*JrbZIP49*) to 84.52 kD (*JrbZIP70*), and the amino acid length of reads ranged from 132 aa (*JrbZIP49*) to 783 aa (*JrbZIP70*).

**Table 1:** Basic information on members of the walnut bZIP transcription factor family

Gene name	Protein ID	Gene ID	Gene length (bp)	Protein length (aa)	CDS length(bp)	PI	MW (Kd)
*JrbZIP1*	XP_018842402.1	109007259	5112	320	963	8.90	35.87
*JrbZIP2*	XP_018850160.1	109012797	7386	320	963	6.39	35.80
*JrbZIP3*	XP_018831015.1	108998773	23704	270	813	9.19	30.08
*JrbZIP5*	XP_018858757.1	109020694	4313	322	969	8.64	36.41
*JrbZIP8*	XP_018809979.2	108982947	4666	415	1248	5.73	45.61
*JrbZIP10*	XP_018805791.1	118,343674	5944	424	1275	9.33	45.87
*JrbZIP11*	XP_018805795.1	108979552	3970	410	1233	9.49	44.30
*JrbZIP12*	XP_018842125.1	109007065	4279	295	888	6.94	32.39
*JrbZIP13*	XP_018811172.2	108983852	3302	444	1335	7.60	47.51
*JrbZIP14*	XP_018811173.2	108983852	3275	434	1305	7.05	46.21
*JrbZIP15*	XP_018813662.1	108985710	6934	429	1290	8.90	46.35
*JrbZIP17*	XP_018847904.1	109011244	1767	227	684	9.74	25.07
*JrbZIP18*	XP_018856740.2	109018988	1711	269	810	9.42	29.29
*JrbZIP20*	XP_018829595.1	108997693	1591	293	882	9.15	32.29
*JrbZIP21*	XP_018829596.1	108997693	1591	293	882	9.87	30.19
*JrbZIP24*	XP_018850039.1	109012706	2924	321	966	6.72	35.39
*JrbZIP26*	XP_018824209.2	108993680	2085	320	963	5.91	36.21
*JrbZIP28*	XP_018821136.1	108991368	4933	578	1737	6.01	63.50
*JrbZIP29*	XP_018848186.1	109011427	11623	337	1014	6.39	37.06
*JrbZIP31*	XP_018839959.1	109005463	5777	430	1293	6.01	46.71
*JrbZIP32*	XP_018815342.1	108986979	4577	579	1740	6.31	63.03
*JrbZIP33*	XP_018841405.1	109006545	3780	570	1713	6.89	62.02
*JrbZIP34*	XP_018821860.1	108991903	3904	352	1059	6.17	39.32
*JrbZIP35*	XP_018828447.1	108996864	3811	393	1182	6.12	42.49
*JrbZIP36*	XP_018835707.2	109002418	3715	387	1164	5.96	41.47
*JrbZIP38*	XP_018829472.1	108997580	6138	357	1074	5.95	37.88
*JrbZIP39*	XP_018835293.2	109002129	14650	413	1242	6.65	43.99
*JrbZIP40*	XP_018852229.1	109014277	7188	408	1227	6.23	43.47
*JrbZIP43*	XP_018821148.1	108991376	1593	155	468	7.99	17.44
*JrbZIP44*	XP_018836880.1	109003262	1549	158	477	8.01	17.76
*JrbZIP45*	XP_018824006.1	108993508	1274	162	489	5.86	18.40
*JrbZIP46*	XP_018858535.1	109020519	1363	157	474	6.97	17.74
*JrbZIP47*	XP_018815338.1	108986974	1490	156	471	6.06	17.36
*JrbZIP48*	XP_018808746.1	108981944	1378	157	474	9.10	17.55
*JrbZIP50*	XP_018808498.1	108981706	1224	145	438	9.21	16.36
*JrbZIP51*	XP_018821766.1	108991829	453	150	453	9.86	16.96
*JrbZIP53*	XP_018853242.2	109015218	882	199	600	5.84	23.09
*JrbZIP54*	XP_018842889.1	109007599	1068	204	615	5.13	23.80
*JrbZIP55*	XP_018816676.1	108988037	806	197	594	5.79	22.70
*JrbZIP56*	XP_018858946.1	109020876	911	196	591	5.75	22.73
*JrbZIP57*	XP_018856866.1	109019104	918	206	621	8.59	23.72
*JrbZIP58*	XP_018816024.1	108987549	489	162	489	5.95	18.89
*JrbZIP59*	XP_018833784.1	109001102	453	150	453	10.04	17.57
*JrbZIP60*	XP_018837265.1	109003548	1408	217	654	9.16	25.21
*JrbZIP61*	XP_018826989.1	108995815	6732	468	1407	5.61	50.84
*JrbZIP62*	XP_018830240.1	108998199	5137	459	1380	5.62	49.69
*JrbZIP63*	XP_018845422.1	109009422	4555	421	1266	4.94	45.95
*JrbZIP64*	XP_018856962.1	109019180	2907	347	1044	5.34	37.71
*JrbZIP68*	XP_018834809.1	109001821	2259	168	507	9.51	18.24
*JrbZIP69*	XP_018840246.1	109005672	1461	205	618	9.09	23.46
*JrbZIP70*	XP_018843647.1	109008121	4598	783	2352	5.76	84.52
*JrbZIP71*	XP_018857669.1	109019758	3302	759	2280	8.42	82.60
*JrbZIP72*	XP_018832980.1	109000523	2632	303	912	4.85	33.95
*JrbZIP73*	XP_018843068.1	109007724	3200	302	909	5.16	33.49
*JrbZIP76*	XP_018817386.1	108988549	1553	270	813	5.46	29.98
*JrbZIP78*	XP_018838122.1	109004136	7895	370	1113	6.24	41.85
*JrbZIP79*	XP_018850850.1	109013266	7522	361	1086	8.36	40.80
*JrbZIP80*	XP_018849635.1	109012452	7810	362	1089	8.46	40.72
*JrbZIP81*	XP_018859508.1	109021341	6552	361	1086	6.82	40.67
*JrbZIP84*	XP_018808536.1	108981744	6336	477	1434	7.30	53.55
*JrbZIP85*	XP_018813854.1	108985859	8372	507	1524	7.05	56.15
*JrbZIP86*	XP_018850960.1	109013359	8791	499	1500	6.39	55.49
*JrbZIP88*	XP_018814127.1	108986066	11455	449	1350	6.69	49.62
*JrbZIP90*	XP_018818230.1	108989164	7516	461	1386	8.35	51.34
*JrbZIP91*	XP_018849403.2	109012303	9132	448	1347	6.44	49.75

### Physicochemical characterization of member proteins of the walnut bZIP transcription factor family

The 91 bZIP transcription factor protein sequences were identified as corresponding to 65 walnut *bZIP* transcription factor family genes (Table 2) using bioinformatic methods such as Hmmer 3.0 combined with the Pfam database and sequence comparison using MEGAX [33]. Based on known sequence information, the online software ExPasy (http://www.expasy.org/), the TMHMM (http://www.cbs.dtu.dk/services/TMHMM/) method, the SignalP3.0 server (http://www.cbs.dtu. dk/services/SignalP) and other online software were employed to predict the affinity, instability coefficients, transmembrane regions, signal peptides, glycosylation sites, phosphorylation sites, and subcellular localization of *bZIP* family genes [34]. The results showed that among the 65 walnut *bZIP* genes, the shortest length was 453 bp, the longest was 23,704 bp, and the CDS length ranged from 438 to 2352 bp. The translated JrbZIP proteins were all between 145 and 783 amino acids in length, with molecular weights ranging from 16.36 to 84.52 KD; the isoelectric points ranged from 4.85 to 10.04 The average hydrophobicity values varied in the range -0.306 ~ -1.158, and the hydrophobicity of all proteins was less than 0, indicating that the JrbZIP proteins were all hydrophilic. Few transmembrane structures were identified; only JrbZIP70, JrbZIP71, JrbZIP72, and JrbZIP73 had transmembrane structures, and 95.6% of the bZIP family protein members were not transmembrane proteins. Relatively few proteins contained signal peptides; only JrbZIP41, JrbZIP45, JrbZIP57, JrbZIP63, and JrbZIP91 contained signal peptides, and 94.5% of the bZIP family protein members were not secretory proteins. Subcellular localization analysis revealed that all JrbZIP proteins localized to the nucleus. Therefore, it was hypothesized that the bZIP proteins of walnut mainly functioned in the nucleus (Table 2).

**Table 2:** Basic information on the walnut bZIP transcription factor protein family

Protein name	Protein ID	Average hydrophobicit	Instability index	Transmembrane structure	Signal peptide	Cell localization
*JrbZIP1*	XP_018842402.1	-0.888	62.64	not have	not have	Nucleus
*JrbZIP2*	XP_018850160.1	-0.831	58.02	not have	not have	Nucleus
*JrbZIP3*	XP_018831015.1	-0.846	51.05	not have	not have	Nucleus
*JrbZIP5*	XP_018858757.1	-0.361	51.85	not have	not have	Nucleus
*JrbZIP8*	XP_018809979.2	-0.572	47.01	not have	not have	Nucleus
*JrbZIP10*	XP_018805791.1	-0.649	48.65	not have	not have	Nucleus
*JrbZIP11*	XP_018805795.1	-0.673	49.69	not have	not have	Nucleus
*JrbZIP12*	XP_018842125.1	-0.679	60.32	not have	not have	Nucleus
*JrbZIP13*	XP_018811172.2	-0.619	58.57	not have	not have	Nucleus
*JrbZIP14*	XP_018811173.2	-0.591	58.47	not have	not have	Nucleus
*JrbZIP15*	XP_018813662.1	-0.656	56.95	not have	not have	Nucleus
*JrbZIP17*	XP_018847904.1	-0.621	61.7	not have	not have	Nucleus
*JrbZIP18*	XP_018856740.2	-0.602	72.77	not have	not have	Nucleus
*JrbZIP20*	XP_018829595.1	-0.591	62.92	not have	not have	Nucleus
*JrbZIP21*	XP_018829596.1	-0.713	61.87	not have	not have	Nucleus
*JrbZIP24*	XP_018850039.1	-0.637	69.79	not have	not have	Nucleus
*JrbZIP26*	XP_018824209.2	-0.879	72.29	not have	not have	Nucleus
*JrbZIP28*	XP_018821136.1	-0.838	57.7	not have	not have	Nucleus
*JrbZIP29*	XP_018848186.1	-0.808	48	not have	not have	Nucleus
*JrbZIP31*	XP_018839959.1	-0.746	62.73	not have	not have	Nucleus
*JrbZIP32*	XP_018815342.1	-0.842	63.8	not have	not have	Nucleus
*JrbZIP33*	XP_018841405.1	-0.842	61.11	not have	not have	Nucleus
*JrbZIP34*	XP_018821860.1	-0.714	59.3	not have	not have	Nucleus
*JrbZIP35*	XP_018828447.1	-0.723	56.64	not have	not have	Nucleus
*JrbZIP36*	XP_018835707.2	-0.765	59.17	not have	not have	Nucleus
*JrbZIP38*	XP_018829472.1	-0.898	48.69	not have	not have	Nucleus
*JrbZIP39*	XP_018835293.2	-0.876	54.55	not have	not have	Nucleus
*JrbZIP40*	XP_018852229.1	-0.914	60.12	not have	not have	Nucleus
*JrbZIP43*	XP_018821148.1	-0.719	53.83	not have	not have	Nucleus
*JrbZIP44*	XP_018836880.1	-0.618	41.26	not have	not have	Nucleus
*JrbZIP45*	XP_018824006.1	-0.756	54.12	not have	have	Nucleus
*JrbZIP46*	XP_018858535.1	-0.725	52.38	not have	not have	Nucleus
*JrbZIP47*	XP_018815338.1	-0.435	59.75	not have	not have	Nucleus
*JrbZIP48*	XP_018808746.1	-0.55	68.1	not have	not have	Nucleus
*JrbZIP50*	XP_018808498.1	-0.552	60.03	not have	not have	Nucleus
*JrbZIP51*	XP_018821766.1	-0.727	55.52	not have	not have	Nucleus
*JrbZIP53*	XP_018853242.2	-0.774	59.98	not have	not have	Nucleus
*JrbZIP54*	XP_018842889.1	-0.91	56.75	not have	not have	Nucleus
*JrbZIP55*	XP_018816676.1	-0.9	76.22	not have	not have	Nucleus
*JrbZIP56*	XP_018858946.1	-0.828	71.15	not have	not have	Nucleus
*JrbZIP57*	XP_018856866.1	-0.806	66.54	not have	have	Nucleus
*JrbZIP58*	XP_018816024.1	-0.852	71.28	not have	not have	Nucleus
*JrbZIP59*	XP_018833784.1	-0.84	53.14	not have	not have	Nucleus
*JrbZIP60*	XP_018837265.1	-0.994	75.78	not have	not have	Nucleus
*JrbZIP61*	XP_018826989.1	-0.786	61.04	not have	not have	Nucleus
*JrbZIP62*	XP_018830240.1	-0.75	60.58	not have	not have	Nucleus
*JrbZIP63*	XP_018845422.1	-0.414	56.48	not have	have	Nucleus
*JrbZIP64*	XP_018856962.1	-0.535	41.36	not have	not have	Nucleus
*JrbZIP68*	XP_018834809.1	-1.135	67.39	not have	not have	Nucleus
*JrbZIP69*	XP_018840246.1	-1.158	56.7	not have	not have	Nucleus
*JrbZIP70*	XP_018843647.1	-0.488	44.4	have	not have	Nucleus
*JrbZIP71*	XP_018857669.1	-0.519	44.39	have	not have	Nucleus
*JrbZIP72*	XP_018832980.1	-0.488	57.75	have	not have	Nucleus
*JrbZIP73*	XP_018843068.1	-0.396	48.17	have	not have	Nucleus
*JrbZIP76*	XP_018817386.1	-0.635	39.12	not have	not have	Nucleus
*JrbZIP78*	XP_018838122.1	-0.436	51.55	not have	not have	Nucleus
*JrbZIP79*	XP_018850850.1	-0.529	49.19	not have	not have	Nucleus
*JrbZIP80*	XP_018849635.1	-0.507	64.8	not have	not have	Nucleus
*JrbZIP81*	XP_018859508.1	-0.476	53.09	not have	not have	Nucleus
*JrbZIP84*	XP_018808536.1	-0.58	65.72	not have	not have	Nucleus
*JrbZIP85*	XP_018813854.1	-0.505	59.34	not have	not have	Nucleus
*JrbZIP86*	XP_018850960.1	-0.496	58.8	not have	not have	Nucleus
*JrbZIP88*	XP_018814127.1	-0.555	57.77	not have	not have	Nucleus
*JrbZIP90*	XP_018818230.1	-0.595	59.57	not have	not have	Nucleus
*JrbZIP91*	XP_018849403.2	-0.468	59.14	not have	have	Nucleus

### Evolutionary tree of the walnut bZIP gene family

**Fig. 1:**
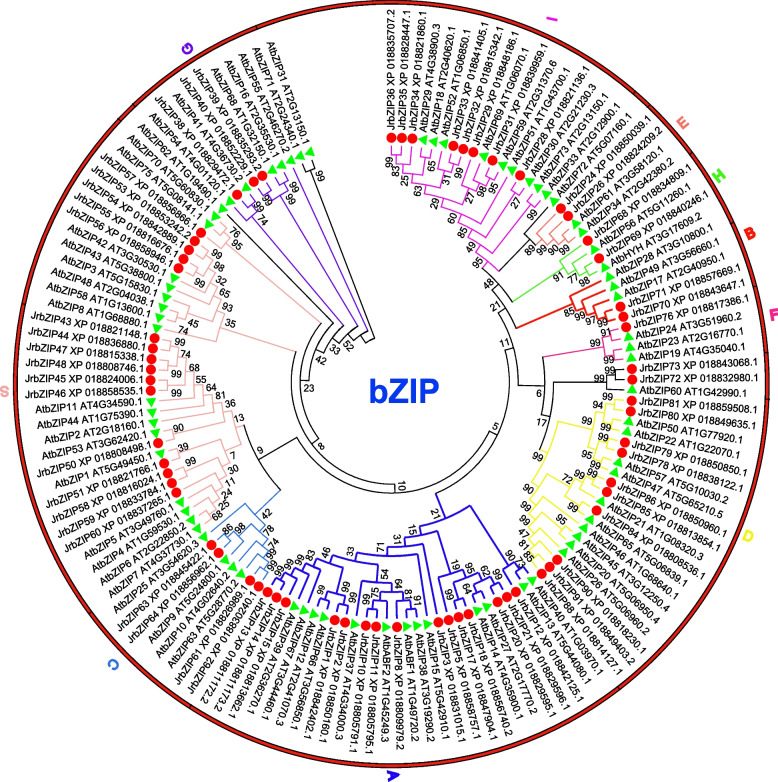
Phylogenetic analysis of bZIP proteins in Arabidopsis thaliana and Juglans regia. Areas of different colors represent different subfamilies. The percentage of replicate trees in which the associated taxa clustered together in the bootstrap test (1000 replicates) is shown next to the branches

To investigate the evolutionary relationships of bZIP family members in walnut and *Arabidopsis*, a maximum likelihood phylogenetic tree of the bZIP family proteins in walnut (65) and *Arabidopsis* (73) was constructed and is shown in Fig. 1. The bZIP family genes of walnut and *Arabidopsis* showed some homology between the two plants. We classified the bZIP gene families in walnut into ten subfamilies, A, B, C, D, E, F, G, H, I, and S, according to the *Arabidopsis* bZIP gene family classification. Among these genes, subfamily S was the largest, with 16 members (showing significant gene duplication and amplification), followed by subfamily A, with 15 members, while subfamily F was the smallest, with only 1 member. Members of subfamilies E and I clustered on the same branch, and members of subfamilies D and F clustered together on another branch, indicating that members of these subfamilies were more closely related.

### Conserved motif and gene structure analyses of the walnut bZIP gene family

**Fig. 2:**
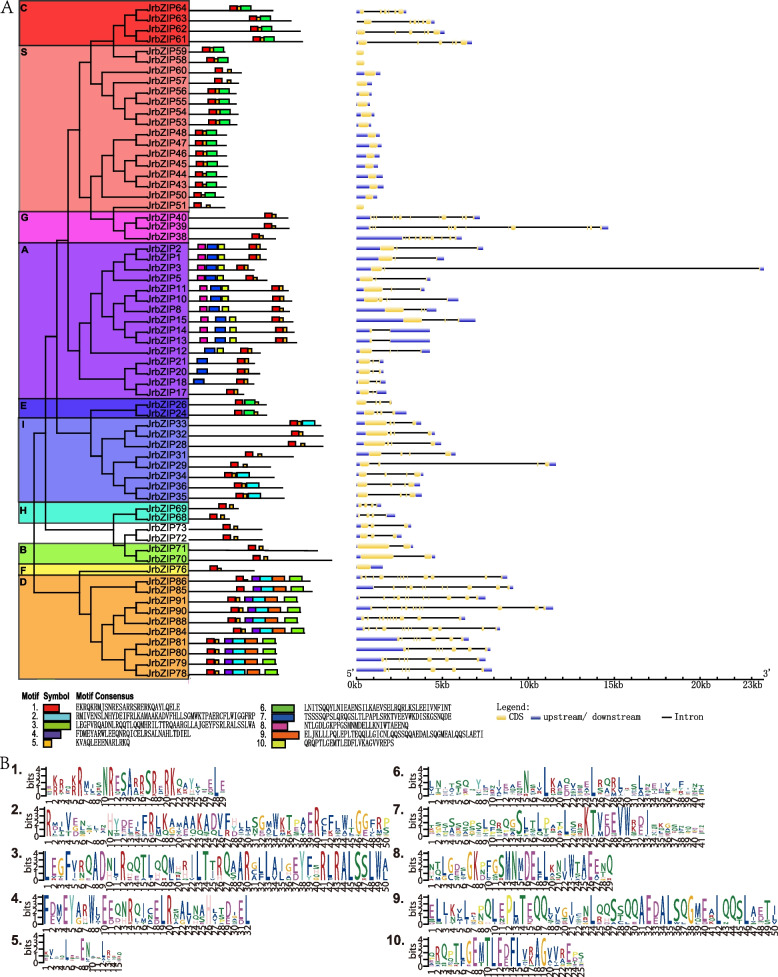
Conserved motif analysis and gene structure analysis of walnut bZIP members. **A** Conservative motif analysis. **B** Gene structure analysis. Number represents branch series. The motifs in the bZIP proteins were identified using MEME. Ten conserved motifs were identified in 65 species and are shown in different colors

To confirm all of the putative *bZIP* gene structures and conserved protein domains [35], we acquired gene exon numbers and positions in the genome as well as motifs and conserved domains of proteins (Fig. 2). We used the online analysis tool Gene Structure Display Server to obtain the gene structure map of bZIP transcription factor family members [36] (Fig. 2A), with the coding region (CDS) in yellow and the 5' noncoding and 3' noncoding regions in the left and right blue regions, respectively. The number of exons in ten families exhibited distinct differences, ranging from 1 to 12. While the members of the G and D subgroups showed 8–12 introns, those of the S and F subgroups had only 1 intron, the B subgroup members had only 1 exon, and the members of the other subgroups presented 3–6 introns. According to our analysis, all of the JrbZIP proteins had motif 1 and motif 5, and the distribution of conserved motifs in the protein sequences of members of the same subgroup was highly similar (Fig. 2B). The motif compositions of subgroups C, S and E were the same, indicating that the functions of different members of the three groups may also be the same.

### Chromosomal analysis and collinearity analysis of walnut *bZIP* members

**Fig. 3:**
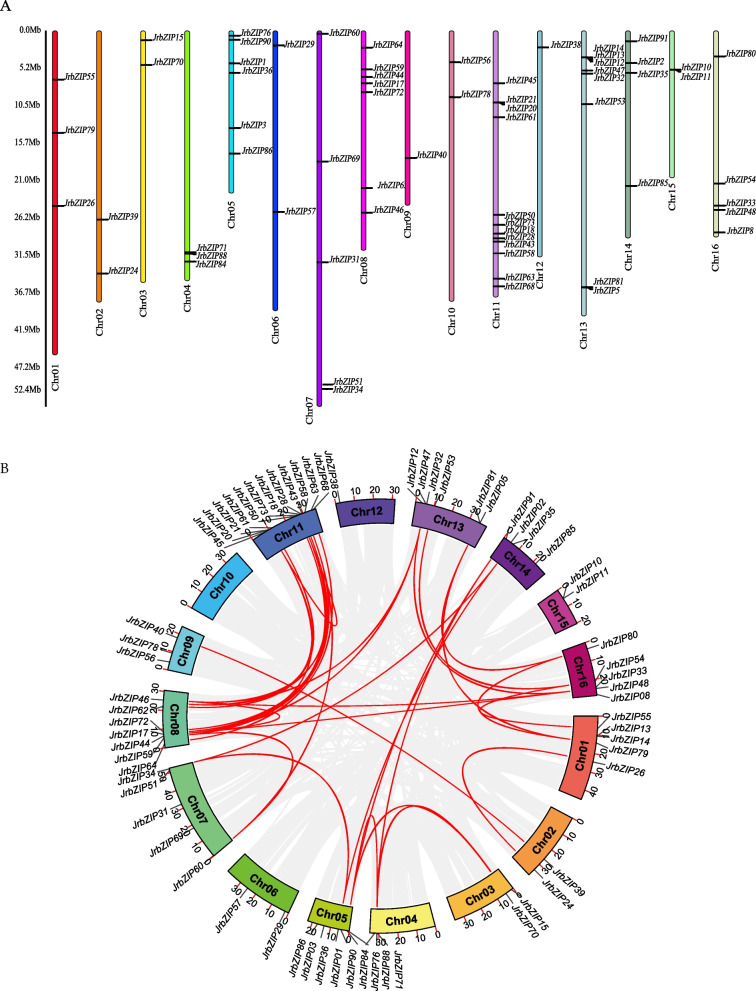
Chromosome mapping and collinearity analysis of the bZIP gene family in walnut. **A** Chromosome mapping. Sixty-five members of the bZIP gene family in walnut are distributed on 16 chromosomes. Different colored regions represent different chromosomes. **B** Collinearity analysis diagram. Fifty-one pairs of collinear genes were found in 65 members of the walnut gene family, and no tandem repeats were found

The positions of the 65 *bZIP* genes on the chromosomes were determined based on their genomic distributions. The results showed that the 65 *bZIP* genes were unevenly distributed on 16 chromosomes (Fig. 3A). Chr11, Chr13, Chr8 and Chr5 contained 12, 8, 7 and 6 *bZIP* genes, respectively, while Chr7 and Chr16 harbored 5 *bZIP* genes each, and the remaining chromosomes contained 1–4 *bZIP* genes.

In the process of evolution, gene families are amplified by means of large segment replication and tandem duplication to improve the adaptability of plants to the environment [37]. In this study, we analyzed the synteny relationships of the bZIP gene family (Fig. 3B) within the walnut genome and identified a total of 51 pairs of fragment duplication events. This situation was considered to indicate large fragment replication, and no tandem duplication events were found [38], suggesting that large fragment replication events were the main driving force for the amplification of the walnut bZIP gene family.

### Differences in *JrbZIP* gene expression in different walnut tissues

**Fig. 4:**
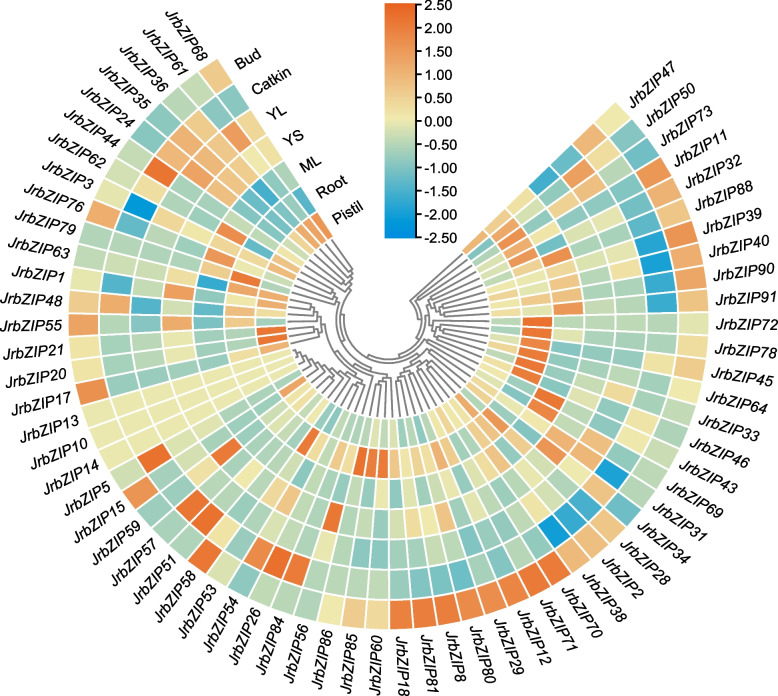
Changes in JrbZIP expression in different tissues. Each value represents the mean ± SE of six biological replicates. (ML: mature leaves; YL: young leaves; YS: young stems.)

We analyzed the expression of 65 *bZIP* genes in seven different tissues of walnut: bud, pistil, catkin, mature leaves, young leaves, yang stem, and root. We obtained the expression levels of *bZIP* genes in each walnut tissue and plotted a heatmap based on the RPKM values of the *JrbZIP* genes (Fig. 4). From the heatmap, it can be seen that the 65 genes were expressed to varying degrees in 7 tissues. Among them, four genes, *JrbZIP24, JrbZIP34, JrbZIP61,* and *JrbZIP73*, presented relatively high expression levels in young leaves (YL), while in mature leaves (ML), they showed relatively low expression levels, indicating that these four genes may participate in the regulation of leaf maturity, as their expression levels decrease with the maturation of walnut leaves. In young stems (YS), the expression levels of six genes, *JrbZIP1, JrbZIP31, JrbZIP34, JrbZIP55, JRbZIP56,* and *JrbZIP59*, were relatively high, suggesting that they may be specifically expressed during the development of young stems.

### Transcriptional expression analysis of bZIP family members in walnut explants under different culture conditions

**Fig. 5:**
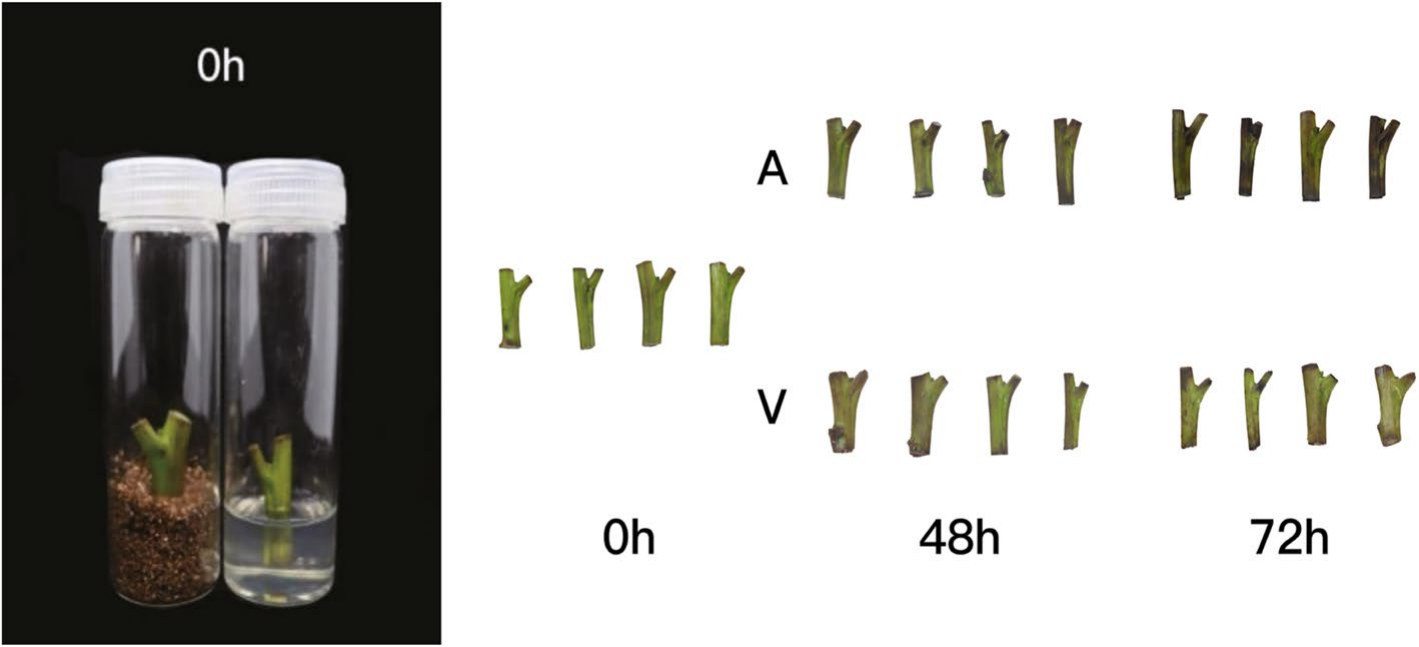
Browning of explants in different media. A: agar treatment, V: vermiculite treatment

**Fig. 6:**
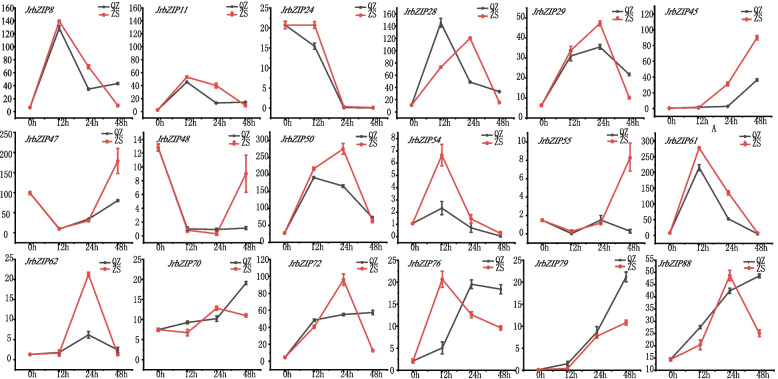
Changes in the expression of JrbZIPs in different media cultures. Each value represents the average ± SE of three biological replicates

Based on the transcriptome analysis of different tissues of walnut, the browning of 'Qingxiang' explants was observed after 12 h, 24 h and 48 h of culture in different media, and it was found that the explants showed significant browning after 48 h of culture in agar medium, while browning was not obvious in vermiculite medium (Fig. 5). The relevant physiological indicators also showed significant differences in the pivotal enzyme activities and total phenolic content of the explants cultured in different medium. (Additional file 8). Further transcriptomic analysis of 'Qingxiang' explants after 12 h, 24 h and 48 h of incubation in both types of media using FPKM as a measure of gene expression showed different degrees of change in all walnut bZIP family gene members (Fig. 6) [39]. Through differential screening, it was found that among 'Qingxiang' *JrbZIP8, JrbZIP11, JrbZIP24, JrbZIP28, JrbZIP29, JrbZIP45, JrbZIP47, JrbZIP48, JrbZIP50, JrbZIP54, JrbZIP54, JrbZIP55, JrbZIP61, JrbZIP62, JrbZIP70, JrbZIP72, JrbZI76, JrbZIP79,* and *JrbZIP88*, 18 of the genes were differentially expressed. The expression of the *JrbZIP55* gene in explants in agar and vermiculite media was not significantly different before 24 h but showed a significant increase in vermiculite after 48 h. The expression of three genes, *JrbZIP70, JrbZIP72* and *JrbZIP88*, showed an increasing and then decreasing trend in vermiculite and a significant increase in agar. These results show that the expression of the four genes differed significantly under the two culture conditions. Additionally, since 'Clear Scent' explants in agar medium gradually began to brown with increasing culture time, it was speculated that these four genes, *JrbZIP55, JrbZIP70, JrbZIP72* and *JrbZIP88*, might be related to the browning of walnut explants. The results were found to be consistent with the transcriptomic data, as verified by real-time quantitative (Additional file 9).

### Cis element analysis and protein interaction prediction

**Fig. 7:**
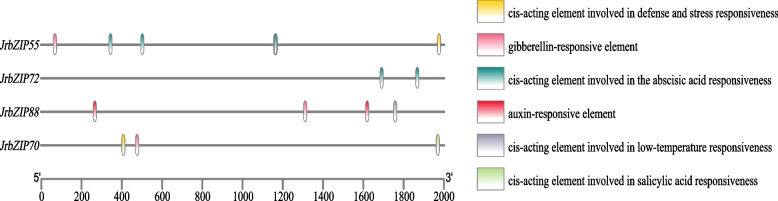
Analysis of the cis-regulatory element of the promoter. Using the Plantcare website, we conducted promoter cis-acting element analysis on the four selected genes, JrbZIP55, JrbZIP70, JrbZIP72, and JrbZIP88. Different colors represent different components

The 2000 bp [40] promoter region elements upstream of the start codons of *JrbZIP55, JrbZIP70, JrbZIP72* and *JrbZIP88* were analyzed by PlantCARE (Fig. 7), and multiple hormone (salicylic acid, abscisic acid, growth hormone and gibberellin) signaling and stress (low temperature, stress and defense) regulatory elements were identified in their promoter regions. Among these regions, *JrbZIP55* contained cis-acting elements involved in gibberellin and abscisic acid reactions and regulatory elements in response to stress and defense; *JrbZIP72* contained only cis-acting elements involved in abscisic acid reactions; *JrbZIP88* contained cis-acting elements involved in growth hormone and gibberellin reactions and regulatory elements involved in the response to low temperature stress; and *JrbZIP70* contained cis-acting elements involved in erythromycin and salicylic acid reactions as well as regulatory elements involved in stress and defense responses.

**Fig. 8:**
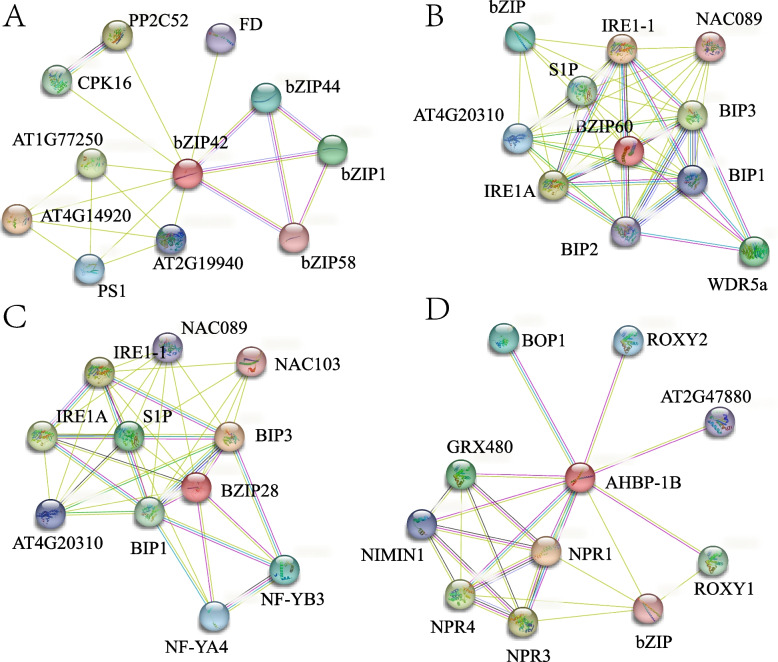
Protein interaction prediction. **A** The walnut bZIP55 protein and Arabidopsis bZIP42 protein are highly homologous proteins. **B** The walnut bZIP72 protein is highly homologous to the Arabidopsis bZIP60 protein. **C** The walnut bZIP70 protein is highly homologous to the Arabidopsis bZIP28 protein. **D** The walnut bZIP88 protein is highly homologous to the Arabidopsis AHBP-1B protein

Thus, the walnut JrbZIP55 protein was identified as being highly homologous to the *Arabidopsis* bZIP42 protein, the walnut bZIP72 protein was identified as being highly homologous to the *Arabidopsis* bZIP60 protein, the walnut bZIP70 protein was identified as being highly homologous to *Arabidopsis* bZIP28, and the walnut bZIP88 protein was identified as being highly homologous to *Arabidopsis* AHBP-1B (Fig. 8). These *Arabidopsis* proteins were used as references for protein interaction prediction. The results showed that the protein highly homologous to walnut JrbZIP55, *Arabidopsis* bZIP42, interacted with the oxidoreductase class AT2G19940 proteins, which are enzymes that act on aldehyde or oxygen-containing group donors, and it is hypothesized that JrbZIP55 is associated with the browning of walnut explants.

## Discussion

Compared to *Arabidopsis* [41] (75 members with an average length of 321 amino acids, divided into 10 subfamilies (A, B, C, D, E, F, G, H, I and G) based on protein structural features), rice [42] (89 members with an average length of 311 amino acids), poplar [43] (86 members, divided into 12 subfamilies based on *Arabidopsis* as a reference), and castor [44] (49 members, divided into 11 subfamilies (I-XI) based on their gene structures, DNA-binding sites, conserved motifs, and phylogenetic relationships), walnut contains 65 bZIP transcription factors with an average length of 463 amino acids, and they have been classified into ten subfamilies (A, B, C, D, E, F, G, H, I and S) using *Arabidopsis* as a reference. In comparison with the conserved motifs found in bZIP transcription factors in other plants, a total of one motif (motif 1) in walnut and *Arabidopsis* and two motifs (motifs 3 and 4) in walnut and poplar were identified, indicating that these additional motifs outside the bZIP domain might be conserved among plant species. However, some additional motifs are variable among species and may be species-specific in plants.

Previous studies showed that *BjubZIP54*, a member of the S2 subfamily of the bZIP family in Capsella [45], was upregulated under both high temperature and drought stresses, presumably to regulate the expression of downstream related genes involved in abiotic stress response. The bZIP family transcription factor *AcePosF21* in kiwifruit [46] mediates the regulation of *AceGP3* expression and *AsA* biosynthesis, thereby reducing oxidative damage caused by cold stress. *PhebZIP47* in moso bamboo [47] positively regulates drought tolerance in transgenic plants by regulating differentially expressed genes (DEGs), including genes in the drought tolerance regulatory pathway and ABA signaling pathway. It is evident that *bZIP* transcription factors regulate downstream related genes as well as the response to various stresses through hormonal signaling in most plants [44, 46]. In this study, based on family analysis and transcriptome data analysis, multiple hormone (salicylic acid, abscisic acid, growth hormone and gibberellin) signaling and stress (low temperature, stress and defense) response elements were also identified in the genes encoding four walnut transcription factors, *JrbZIP55, JrbZIP70, JrbZIP72* and *JrbZIP88*, by promoter cis-acting element analysis, as were reciprocal protein prediction regulatory elements. It is hypothesized that bZIP genes may act as transcriptional regulators involved in stress response regulation in walnut plants.

The browning of plants after damage occurs mainly due to a decrease in the ascorbic acid content and increases in phenylalanine deaminase and lipoxygenase activities [48]. Some studies have found that there was a strong linear relationship between total phenolic compound content of microshoots and increasing antioxidant activity [26]. In contrast, *AT2G19940* is present in the *Arabidopsis* chloroplast stroma as a donor reductase that acts on aldehydes or oxidative groups as an acceptor to participate in the amino acid metabolic synthesis process with NAD and NADP as acceptors [49]. In this study, AT2G19940 and AtbZIP42 were predicted to interact with each other. *PgbZIP131* in ginseng [50] belongs to the S subfamily, which shows a strong response to abiotic stresses, where *AtbZIP44* plays an important role in drought stress [51] and belongs to the same subfamily as AtbZIP42. Transgenic *Arabidopsis thaliana* overexpressing *SibZIP8* from cereals [52] shows a higher germination rate under salt stress than the wild type. AtbZIP42 in *Arabidopsis* is the only protein that shows reasonable homodimeric activity in yeast [53]. AtbZIP42 and JrbZIP55 are homologous proteins, and it is speculated that the JrbZIP55 transcription factor may regulate the browning of walnut explants. Concerning the specific role the *JrbZIP55* gene plays in walnut browning and which downstream structural genes are regulated to cause alterations in synthetic pathways that affect walnut exine browning, further elucidation of the molecular mechanisms of these specific regulatory syntheses involved in plant water stress is needed.

## Conclusion

Sixty-five bZIP genes were identified in the walnut genome, distributed on sixteen chromosomes and divided into ten subfamilies. The expression of four genes, *JrbZIP55, JrbZIP70, JrbZIP72,* and *JrbZIP88*, showed significant differences in different media, and there were multiple signaling and regulatory elements in the promoter regions of these genes. The walnut JrbZIP55 protein and *Arabidopsis* bZIP42 protein are highly homologous, and the proteins interacting with *Arabidopsis* bZIP42 include the AT2G19940 oxidoreductases, which act on aldehyde or oxygen-containing donors. This suggests that *JrbZIP55* may be involved in the regulation of walnut explant browning.

## Methods

### Plant materials and treatment

The test material 'Qingxiang' walnut (*Juglans regia L.*) was grown in the specimen garden of Hebei Agricultural University, rooted from cuttings, and was used at 6 years of age, with normal growth and fruiting. The identification of the 'Qingxiang' cultivar was performed by the Hebei Provincial Forestry Species Validation and Approval Committee in 2003, with accession number Ji S-ETS-JR-007–2003. No special permission was necessary to collect such samples. In mid-April, developing branches of uniform thickness and robust growth were collected from the middle and upper parts of the trees, cut into 3 cm stem segments, with each segment containing 1 or 2 full buds, and subsequently washed in the following sequence: laundry detergent water, water, 75% alcohol for 30 s for surface sterilization, 0.1% HgCl_2_ for 8 min for sterilization, and sterile water (6 times), after which they were transferred to vermiculite and agar medium containing DKW [54]. The materials were then grown at 25°C under a light intensity of 3000 lx and a 16 h light and 8 h dark cycle. Samples were collected at 0 h, 12 h, 24 h, and 48 h and snap frozen in liquid nitrogen for RNA-Seq. Seven different tissues were taken from leaf buds, female flowers, male flowers, mature leaves, young leaves, roots and fruits at different times of the growing season, with the older leaves taken on July 15 and the other six tissues taken on April 23. Three stem sections were mixed as a single sample, and three replicates were performed. RNA-Seq was carried out by Novogene Co. The experimental method of this study is shown in a flowchart (Additional file 10).

### Identification and bioinformatics analysis of the bZIP gene family in walnut

Walnut whole-genome data were downloaded from the NCBI database (http://www.ncbi.nlm.nih.gov/. Accessed 20 May 2023). The hidden Markov model files for bZIP transcription factors were downloaded from the Pfam database (http://pfam.xfam.org/. Accessed 20 May 2023), and these hidden Markov model files were used as search criteria. A program in hmmer3.0 software was used to search for walnut protein sequences (E ≤ 1 × 10 − 10), the obtained results were deduplicated, and extraction was performed by using the SMART (http://smart.embl-heidelberg.de/. Accessed 20 May 2023) and NCBI-CDD (https://www.ncbi.nlm.nih.gov/Struc-ture/cdd/wrpsb.cgi. Accessed 21 May 2023) databases for further identification and screening, and the walnut bZIP family protein sequences were finally obtained (Additional file 2) [55]. The isoelectric point and molecular weight of walnut bZIP protein sequences were analyzed using the online tool ProtParam (https://www.expasy.org/protparam/. Accessed 21 May 2023).

We used the alignment result to construct a phylogenetic tree via the neighbor-joining method in MEGA7 software (http://www.megasoftware.net/. Accessed 22 May 2023). GSDS v2.0 (http://gsds.cbi.pku.edu.cn/. Accessed on 26 May 2023) was used to predict the inline-exon structure of the bZIP gene family in walnut [56]. MEME (http://meme-suite.org/tools/meme. Accessed on 26 May 2023) was used to analyze the motif composition of JrbZIP proteins (motif size setting: 6–50, number setting: 20, default values for the other parameters), and the results were visualized using the program “Gene Structure View (Advanced)” in TBtools software. The protein sequences of walnut were downloaded from GenBank (accession GCA 001411555.2). The types, numbers and functions of the cis-acting elements of the walnut *bZIP* gene promoters were analyzed using the PlantCARE (http://bioinformatics.psb.ugent.be/webtools/plantcare/html/. Accessed on 26 May 2023) website. Finally, the results were visualized using the program “Simple Biosequence Viewer” from TBtools software. Information on the chromosomal position of each *JrbZIP* gene was obtained from the walnut gene GFF file, and duplication events in the genome were analyzed using MCScanX software (Additional file 5). The chromosomal localization of the walnut bZIP gene family was determined using the TBtools software program (Gene Location Visualize from GTF/GFF).

### Physicochemical analysis of walnut JrbZIP protein

The isoelectric point and molecular weight of walnut bZIP protein sequences were analyzed using the online tool ProtParam (https://www.expasy.org/protparam/**.** Accessed on 26 May 2023). The online software Plant-mPLoc 2.0 (http://www.csbio.sjtu.edu.cn/bioinf/plant/. Accessed on 26 May 2023) was used to predict and analyze the subcellular localization of walnut bZIP proteins. Protein interaction analysis was performed using STRING (https://string-db.org/cgi/input.pl. Accessed on 29 May 2023).

### Transcriptional expression analysis of the JrbZIP gene in different tissues of walnut

We analyzed the expression of walnut *bZIP* genes in seven different tissues: buds, female flowers, male flowers, mature leaves, young leaves, roots, and fruits. We also used RNA-Seq data to screen differentially expressed genes using FPKM values as a gene expression measure and padj < 0.05 and log2(golden change > 0) as thresholds for screening differential expression. Data were also visualized using TBtools software to illustrate the content of walnut bZIP genes in buds, female flowers, male flowers, mature leaves, young leaves, roots, and fruits. The sequence data were deposited in GSA (https://bigd.big.ac.cn/gsa/browse/CRA011462).

### Transcriptional expression analysis of the JrbZIP gene in walnut explants

We analyzed the genes that were specifically expressed in stem segments based on RNA-Seq data from different tissues and analyzed the changes in the expression of bZIP genes associated with explants cultured in different media for 0 h, 12 h, 24 h and 48 h based on RNA-Seq explant data. The sequence data were deposited in GSA (https://bigd.big.ac.cn/gsa/browse/CRA011411).

We used a kit extraction method for RNA extraction. The kits used for RNA extraction and DNA extraction were from Tiangen (product numbers: DP441 and DP350, respectively). The reagents used for reverse transcription, PCR and real-time PCR were from Takara (product numbers: RR047A, RR901A and RR820A, respectively). For RNA isolation experiments, all samples were immediately frozen in liquid nitrogen and extracted using a Tiangen Plant RNA Extraction Kit according to the manufacturer’s protocol. First-strand cDNA was synthesized with a Tiangen Inverse Transcription Kit [57].

## Supplementary Information

**Additional file 1: **bZIP protein sequence of *Arabidopsis thaliana*.

**Additional file 2: **bZIP protein sequence of *Juglans regia*.

**Additional file 3: **bZIP CDS Sequence of *Juglans regia.*

**Additional file 4:** bZIP gene sequence of *Juglans regia.*

**Additional file 5: **Collinearity data of JrbZIP gene family.

**Additional file 6: Table S1.** Information  of JrbZIP gene family.

**Additional file 7: Table S2.** Primers used in real-time PCR.

**Additional file 8: Figure S1.** Enzymatic activities of explant during culture in different medium.

**Additional file 9: Figure S2.** Changes in the expression of some genes.

**Additional file 10: Figure S3.** Experimental method flowchart.

## Data Availability

The RNA-Seq datasets are available in the GSA (submission number: CRA011411, https://bigd.big.ac.cn/gsa/browse/CRA011411; submission number: CRA011462, https://bigd.big.ac.cn/gsa/browse/CRA011462). The CDS and genome sequences of bZIPs in walnut were retrieved from the whole walnut genome database (accession GCA_001411555.2) in NCBI. All data and materials are presented in the main paper and additional file.

## Abbreviations

BZIP: Basic Leucine Zipper
V: Vermiculite-DKW medium
A: Agar-DKW medium
DEGs: Differential gene expression
ML: Mature leaves
YL: Young leaves
YS: Young stems
