# Supplementary material for: The role of walnut bZIP genes in explant browning
Source: BMC Genomics. 2023 Jul 5;24:377. doi: 10.1186/s12864-023-09492-1 (PMC10324250; doi:10.1186/s12864-023-09492-1)
Supplement: Supplementary file 8 — Additional file 8: Figure S1. Enzymatic activities of explant during culture in different medium. [file 12864_2023_9492_MOESM8_ESM.pdf]

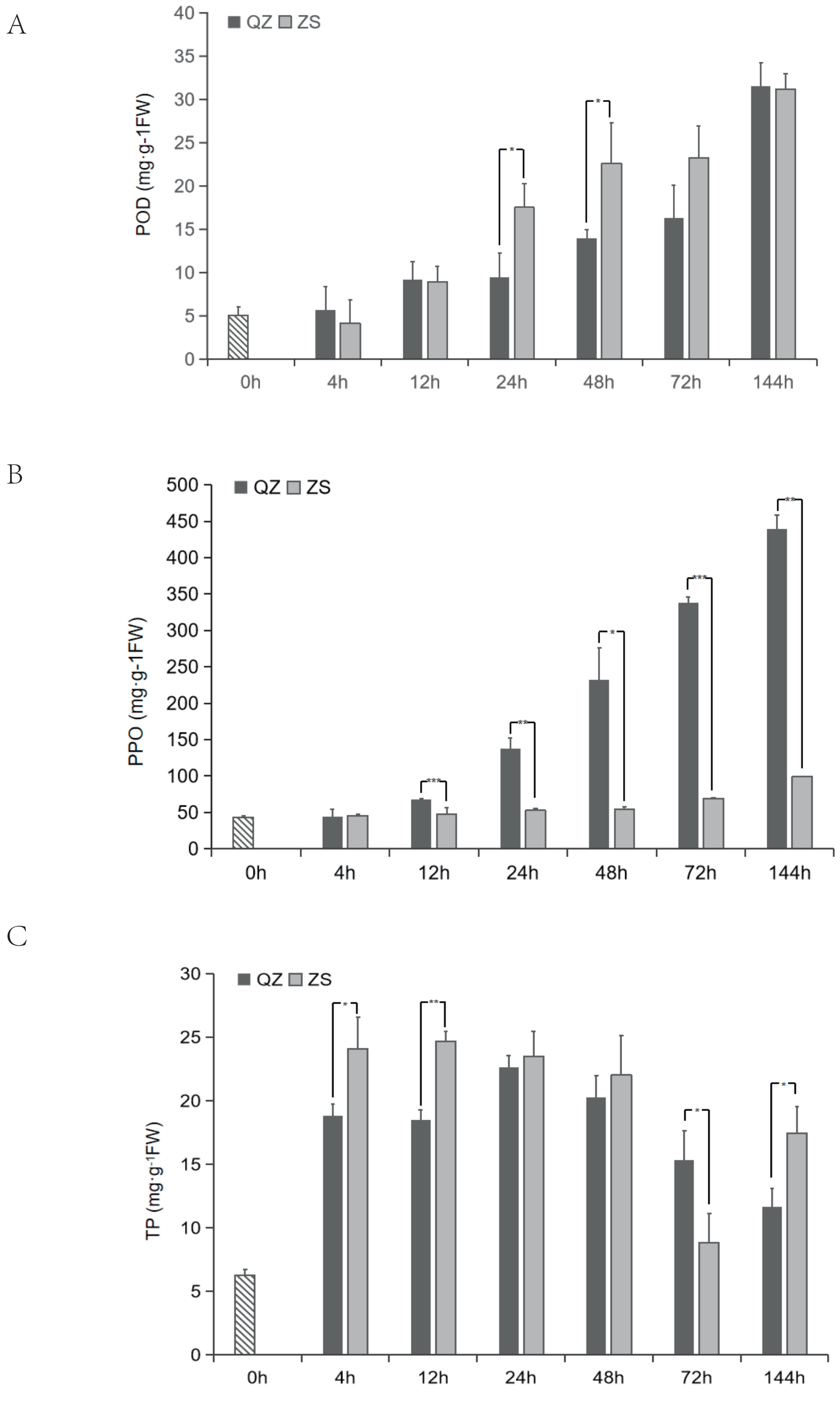

Figure S1 Enzymatic activities of explant during culture in different medium.
A: POD; B: PPO; C: total phenol (TP)
