# Supplementary figures and images for: The role of walnut bZIP genes in explant browning
Source: BMC Genomics. 2023 Jul 5;24:377. doi: 10.1186/s12864-023-09492-1 (PMC10324250; doi:10.1186/s12864-023-09492-1)

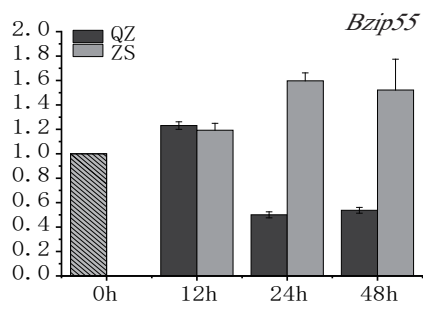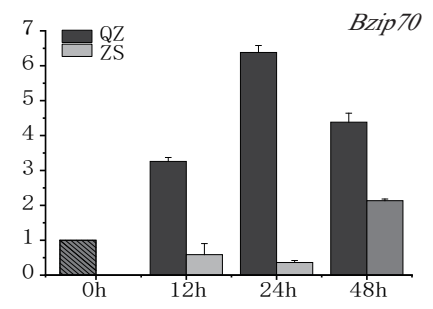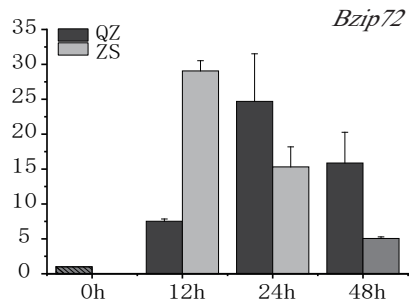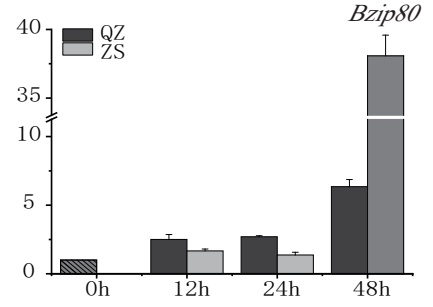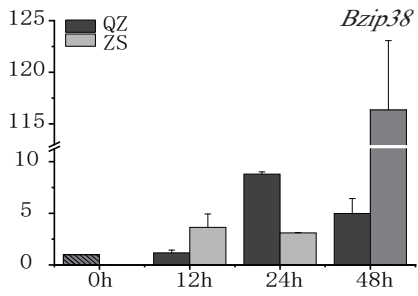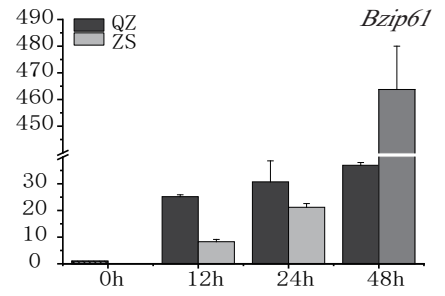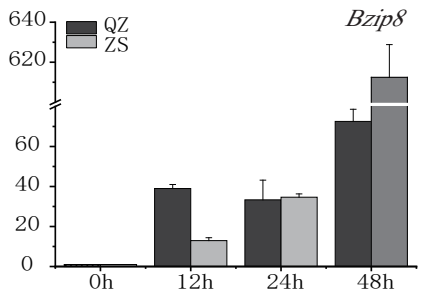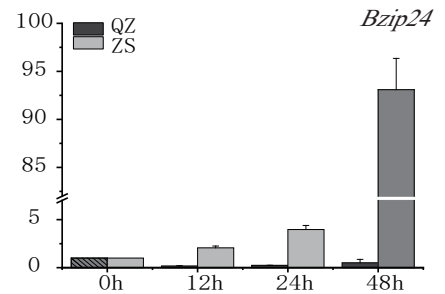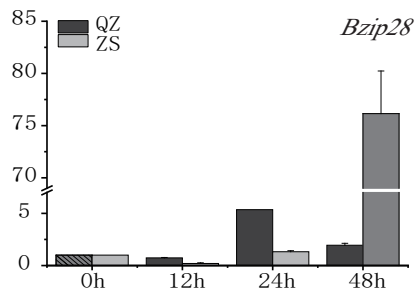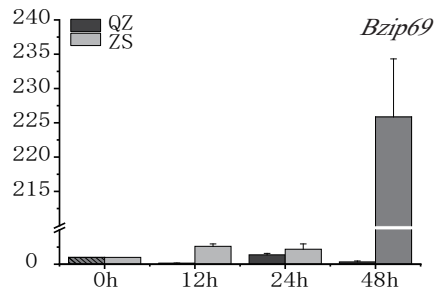

Figure S2 Changes in the expression of some genes.

Supplement: Supplementary file 9 — Additional file 9: Figure S2. Changes in the expression of some genes. [file 12864_2023_9492_MOESM9_ESM.pdf]

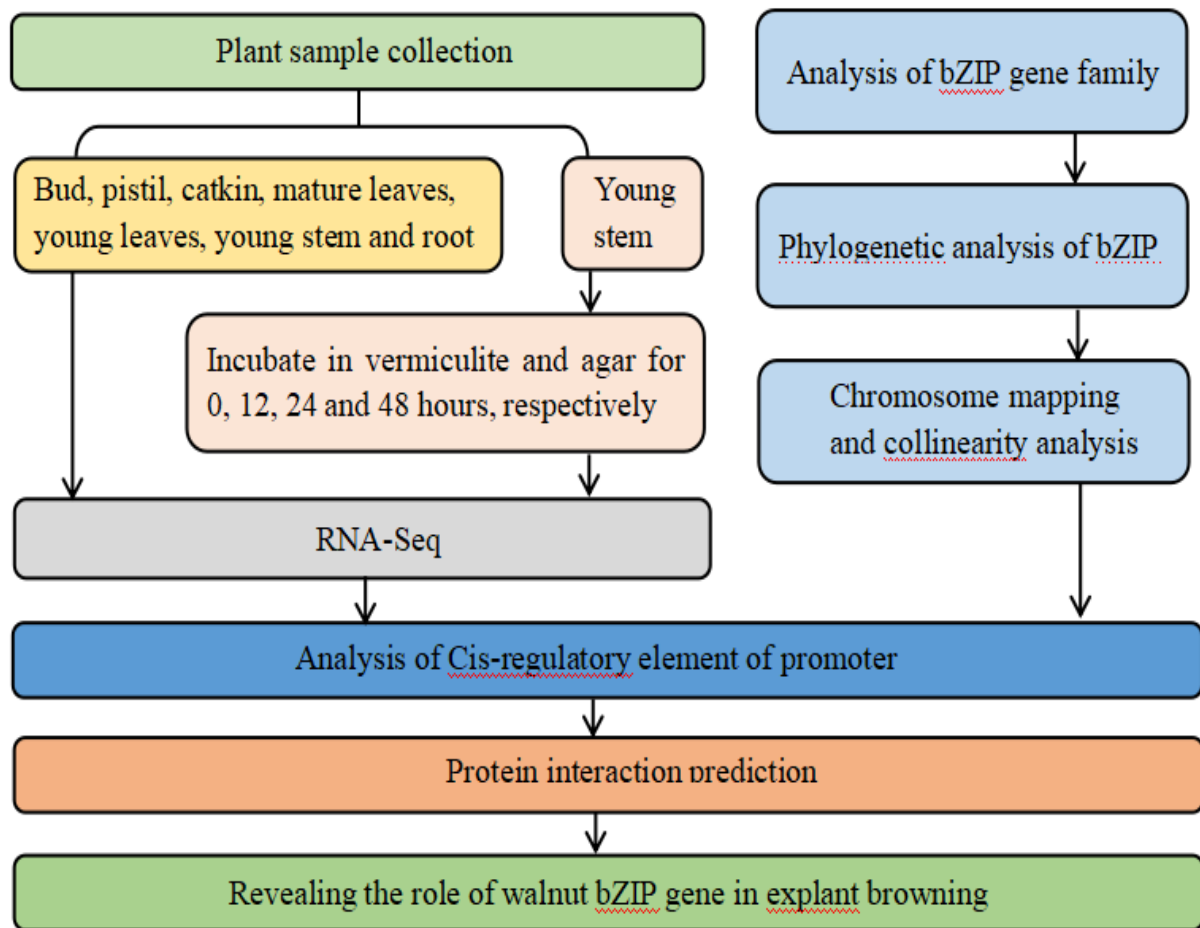

Figure S3. Experimental method flowchart

Supplement: Supplementary file 10 — Additional file 10: Figure S3. Experimental method flowchart. [file 12864_2023_9492_MOESM10_ESM.pdf]
